# The Teaching of Pathology at St. Bartholomew's Hospital

**Published:** 1911-12-16

**Authors:** 


### The Hospital

The Workers' Newspaper of Administrative Medicine and Institutional Life, Administration, National Insurance and Health.

No. 1327, Vol. LI. SATURDAY, DECEMBER 16TH, 1911. PRICE ONE PENNY.

### THE TEACHING OF PATHOLOGY AT ST. BARTHOLOMEW'S HOSPITAL.

An interesting scheme is in prospect at St. Bartholomew's Hospital which should have an important influence on the teaching of pathology in that institution. To every physician and surgeon in charge of wards a pathological clerk is to be appointed, whose duty it will be to attend every morning in the pathological laboratory, and there to examine any material received from the wards to which he is attached. He will work under the supervision of the demonstrator, and all reports will be countersigned by the demonstrator.

The question as to the best method of teaching pathology has always been a difficult one. The student, when he has finished his anatomy and physiology, is confronted by a large number of subjects which are for the most part new to him. It is by no means easy for him to realise that these are all closely dependent on one another; rather is he inclined to regard each subject as a separate entity, having no bearing on any of the others. As the curriculum is a very full one, and the student is usually pressed for time, he is apt to devote most of his attention to those subjects which seem likely to be of obvious use to him in his future practice; the remainder are more or less neglected. One of the subjects which too often goes to the wall in this way is pathology, and in our opinion this is largely due to the manner in which this subject is taught in many medical schools at the present day.

Every examining body requires the student to attend a course of lectures and demonstrations on pathology, but the time allotted to these is barely sufficient for a hurried survey of so wide a subject, and the instruction given in practical pathology and bacteriology is even less adequate. There are, of course, some who pursue the subject with enthusiasm, but we are speaking of the average student, and it is for him that every system of education must be devised. If this state of affairs is to be remedied pathology must no longer be treated as a detached and isolated subject. The student must be shown that it is not merely a theoretical topic of purely academic interest, but is intimately connected with the practical side of his work.

We should like to see every lecturer in medicine, surgery, and midwifery commencing the study of a disease with a full description of its pathology, and impressing on the student the fact that without a thorough grasp of this he can never appreciate properly either the significance of symptoms or the efficacy of treatment; and the same course should be followed in all clinical teaching. Then again, all clerks and dressers should be encouraged, or even required, to follow up the history of their cases from the wards to the pathological laboratory. Let the clerk assist at the investigation of all material sent from his wards; let the dresser assist at the examination, both by the microscope and by the naked eye, of all tumours removed from cases under his charge. At present the wards and the pathological laboratory are too much cut off one from the other. The workers in the laboratory seldom see the cases on which they are asked to report, while the clerks and dressers, and sometimes even the physicians and surgeons, are often quite ignorant of the reasoning on which these reports are based, and are apt to regard them as coming almost from another world which is beyond their ken. It may be objected that an incursion of clerks and dressers would interfere with the work of the laboratory, but if their attendance were confined within reasonable hours, we do not believe this would be the case. Moreover, in some hospitals such attendance is already required, though we are afraid that the rule is more honoured in the breach than in the observance. We believe that some scheme such as we have sketched out would go far to remove the student's indifference towards pathology. It would teach him that pathology is inseparably interwoven with all medicine, that a sound knowledge of it will do more than anything else to help him to solve difficulties of diagnosis and treatment, and that nearly all the recent advances in medical and surgical practice have sprung directly from pathological research. Some years ago the University of Cambridge constituted pathology a special subject, to be taken at a separate part of the M.B. examination. This change was all to the good, but the new system remains open to the same objection that it rather confirms the separation of pathology from medicine and surgery, and obscures its great practical value. The authorities of St. Bartholomew's Hospital are to be congratulated on their new departure. If other hospitals will follow their example, something will have been done to remove the common reproach that, of the vast amount of pathological material available in London and the large provincial towns, a great proportion is wasted.

